# Microbiological profile and epidemiological perspective on urinary tract infections (UTIs) in a tertiary medical center in Western Mexico

**DOI:** 10.3389/fmicb.2026.1734551

**Published:** 2026-01-29

**Authors:** Pedro Reyes-Martinez, Erick Sierra-Diaz, Pablo Cesar Ortiz-Lazareno, Mariana Garcia-Gutierrez, Adrián Ramírez-de-Arellano, Elena Sandoval-Pinto, Rosa Cremades

**Affiliations:** 1 Departamento de Fisiología, Centro Universitario de Ciencias de la Salud, Universidad de Guadalajara, Guadalajara, Jalisco, Mexico; 2 Departamento de Clínicas Quirúrgicas, Centro Universitario de Ciencias de la Salud, Universidad de Guadalajara, Guadalajara, Jalisco, Mexico; 3 División de Epidemiología, UMAE Hospital de Especialidades Centro Médico Nacional de Occidente, Guadalajara, Jalisco, Mexico; 4 División de Inmunología, Centro de Investigación Biomédica de Occidente, Guadalajara, Jalisco, Mexico; 5 Escuela de Medicina y Ciencias de la Salud Tec de Monterrey Campus Guadalajara, Guadalajara, Jalisco, Mexico; 6 Instituto de Investigación en Cáncer e Infecciones, Departamento de Microbiología y patología, Centro Universitario en Ciencias de la Salud, Universidad de Guadalajara, Guadalajara, Jalisco, Mexico; 7 Departamento de Biología Celular y Molecular, Centro Universitario de Ciencias Biológicas y Agropecuarias, Universidad de Guadalajara, Zapopan, Jalisco, Mexico; 8 Departamento de Microbiología y Parasitología, Centro Universitario en Ciencias de la Salud, Universidad de Guadalajara, Guadalajara, Jalisco, Mexico

**Keywords:** antibiotics, healthcare-associated infections, microbiological profile, resistance, urinary tract infections

## Abstract

**Background:**

Urinary tract infections are among the most frequent healthcare-associated infections and represent a major public health challenge due to their increasing antimicrobial resistance. Data from tertiary-care hospitals in Mexico remain scarce. This study aims to describe prevalence and microbiological profile of healthcare-associated UTIs in a tertiary medical facility in western Mexico.

**Methods:**

A cross-sectional study included all UTI cases recorded from January to December 2024. Data was obtained from the institutional epidemiological surveillance platform (INOSO). Descriptive statistics were applied using measures of central tendency, proportions and confidence intervals. Antimicrobial resistance profiles were analyzed using automated Vitek® testing for Antibiotic susceptibility testing and mass spectrometry for pathogen identification.

**Results:**

A total of 376 patients were included (mean age 52.9 years; 53.2% women). Healthcare-associated UTIs represented 80.6% of cases. Monthly incidence displayed a multimodal pattern with peaks in April and October. Nephrology, Cardiology, and Neurosurgery accounted for >50% of cases. Among 120 isolates, bacteria comprised 70.8%, mainly *Escherichia coli* (35.8%), *Klebsiella pneumoniae,* and *Pseudomonas aeruginosa*; fungal isolates (29.2%) were predominantly *Candida albicans*. Extensive drug resistance was observed in *Providencia rettgeri* (resistant to all tested antibiotics) and *Acinetobacter baumannii.*

**Conclusion:**

UTIs in a tertiary-care hospital in Mexico exhibit high prevalence, multimodal temporal dynamics, and alarming antimicrobial resistance. Continuous surveillance, antimicrobial stewardship, and targeted infection-control strategies are urgently needed in high-risk hospital units.

## 1 Introduction

Healthcare-associated infections (HAIs) are considered one of the greatest threats to patient safety worldwide. Developed countries such as the US, UK, Germany, Australia and others have a robust healthcare system and cutting-edge medicine. Part of its success lies in the rapid epidemiological transition. In other words, infectious diseases have been largely eradicated, and the US has implemented sufficiently successful prevention strategies for chronic degenerative diseases. However, despite these resources, HAIs remain a major problem, as they are the sixth leading cause of death, surpassing traffic accidents and HIV infections. In 2002, approximately 1.7 million HAIs and 99,000 deaths were reported. Estimated costs of an average of $31 billion, of which $28.5 billion could have been saved by implementing appropriate prevention strategies (Liu and Dickter, 2020).

A study conducted in the United States, which included a total of 183 hospitals, reported a HAI frequency of 4% in hospitalized patients. This implies that 648,000 patients experienced a total of 721,800 HAIs (Magill et al., 2014). The working group reported a decrease to 3.2% (*p* < 0.001) by 2015. The authors concluded that implementing specific HAI prevention programs is a good alternative for reducing frequency (Magill et al., 2018). In 2016, a retrospective study analyzed HAI-related mortality and hospital stay over a six-year period (2006–2012) and reported no significant changes in these indicators (Glied et al., 2016). The above aligns with our initial proposal; that is, despite having a tangible problem and expenses that totaled an additional cost of $10,000 per patient, a specific prevention strategy is not actually in place.

In 2017, the WHO reported that adverse events such as HAIs occurred in one out of every ten patients receiving medical care, with pneumonia and surgical site infections being the most frequent. By 2018, HAIs ranked third as the most frequent adverse event worldwide in hospitalized patients (Schwendimann et al., 2018; World Health Organization, 2022).

Another important implication related to HAIs is mortality. Due to the complexity of this public health issue globally, it is difficult to accurately establish specific mortality rates. In 2019, a published study revealed that in the United States and countries of the European Union, 671,689 cases of infections caused by antibiotic-resistant organisms occurred during 2015. The reported mortality rate was 33,110 cases. The above data implies an incidence of 131 cases per 100,000 inhabitants and a specific mortality rate of 6.44 deaths per 100,000 inhabitants (Cassini et al., 2019).

The most common and studied healthcare-associated infections (HAIs) are ventilator-associated pneumonia (VAP), catheter-related bloodstream infection (CRBSI), surgical site infection (SSI), and catheter-associated urinary tract infection (CAUTI). Previous studies have reported prevalence rates in tertiary care centers in Mexico (Sierra-Diaz et al., 2024; Eb-Rejón et al., 2025).

The role of the urinary tract infections (UTIs) in the HAIs ground is among the most significant public health problems worldwide. They constitute one of the most common infectious diseases and rank among the leading causes of outpatient consultations and general morbidity in Mexico, affecting millions annually. The situation has worsened due to increasing antibiotic resistance, particularly among hospitalized patients. Although national statistics often aggregate all levels of healthcare, a considerable proportion of complicated infections are treated in tertiary-care hospitals (Pasillas Fabian et al., 2021; Peck and Shepherd, 2021; Ortega-Lozano et al., 2023).

Tertiary medical centers in Jalisco act as referral hubs managing complex UTI cases—such as pyelonephritis, renal abscesses, urosepsis, and infections refractory to outpatient therapy. In these facilities, UTI prevalence reaches critical levels, particularly among patients with comorbidities or severe complications. These hospitals are focal points for multidrug-resistant pathogens, emphasizing the need to understand the magnitude of the problem to improve prevention and therapeutic strategies (Pasillas Fabian et al., 2021; Peck and Shepherd, 2021; Ortega-Lozano et al., 2023; Medina and Castillo-Pino, 2019).

Determining hospital UTI prevalence enables the identification of at-risk populations, monitoring of antimicrobial resistance patterns, and prevention of healthcare-associated infections. However, accurate incidence estimation remains challenging due to diagnostic variability and underreporting. Medina and Castillo-Pino (2019) observed a bimodal age distribution with a primary peak among women aged 14–25 years. In 2023, the institutional CAUTI rate was 4.2 per 1,000 catheter-days, with resistant strains posing major challenges. In Jalisco, UTI prevalence follows national patterns.

The present research aims to describe frequency and the role of UTIs as HAIs and its microbiological profile in one of Mexico’s largest tertiary-care hospitals.

## 2 Methods

A cross-sectional study was conducted. Demographic and microbiological data for patients were obtained from the INOSO surveillance platform of the Mexican Social Security Institute (IMSS) during the period from January to December 2024. A database was constructed to analyze pathogen frequency and antimicrobial resistance patterns.

Microbial identification was performed using matrix-assisted laser desorption/ionization-time-of-flight mass spectrometry (MALDI-TOF MS) with the VITEK® MS PRIME system (bioMérieux, Marcy-l’Étoile, France). Antimicrobial susceptibility testing (AST) was performed using the automated VITEK® 2 system (bioMérieux, Marcy-l’Étoile, France), which determines minimum inhibitory concentrations (MICs) using standardized automated methods. Susceptibility results were interpreted according to the Clinical and Laboratory Standards Institute (CLSI) guidelines. Demographic data and frequencies were calculated using conventional descriptive methods, including measures of central tendency, proportions, and confidence intervals, with Microsoft Excel V16.7® (Microsoft Corporation, Redmond, WA, USA) and Open Epi (Open-Source Epidemiological Statistics for Public Health, Bill & Melinda Gates Foundation, Emory University, Atlanta, GA, USA). Graphs and figures were created in R (version 4.4.1) using RStudio. No artificial intelligence was used in this study.

The study was approved by the local Ethics and Research Committees, with registration number R-2023-1301-189/COFEPRIS 17 CI 14039114/COMBIOETICA 14 CEI 30290123.

## 3 Results

### 3.1 Demographic characteristics

**Figure 1:**
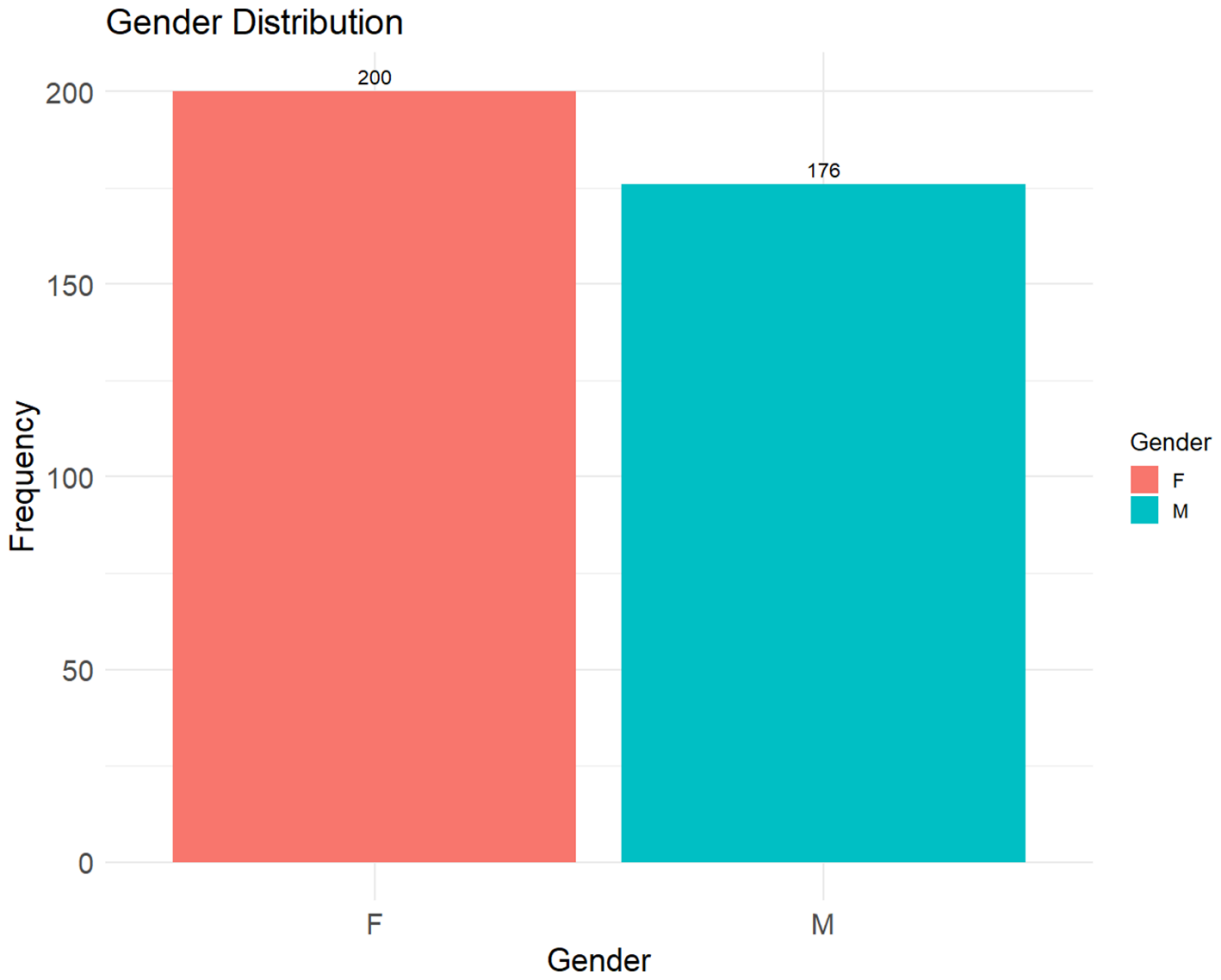
Gender distribution. Distribution of urinary tract infection cases by sex. The bar chart illustrates the frequency of cases among the 200 female and 176 male patients included in the study.

The study included 376 patients with a mean age of 52.9 years (CI95% 50.74–55.03). The cohort comprised 200 women (53.2%) and 176 men (46.8%). Details are depicted in Figure 1. Regarding infection classification, 303 cases (80.6%) were classified as CAUTIs, while 73 (19.4%) were classified as non-catheter-associated UTIs. Microbiological confirmation was available for a subset of cases, as detailed below.

### 3.2 Relationship between patient age and the duration of healthcare-associated infection

**Figure 2:**
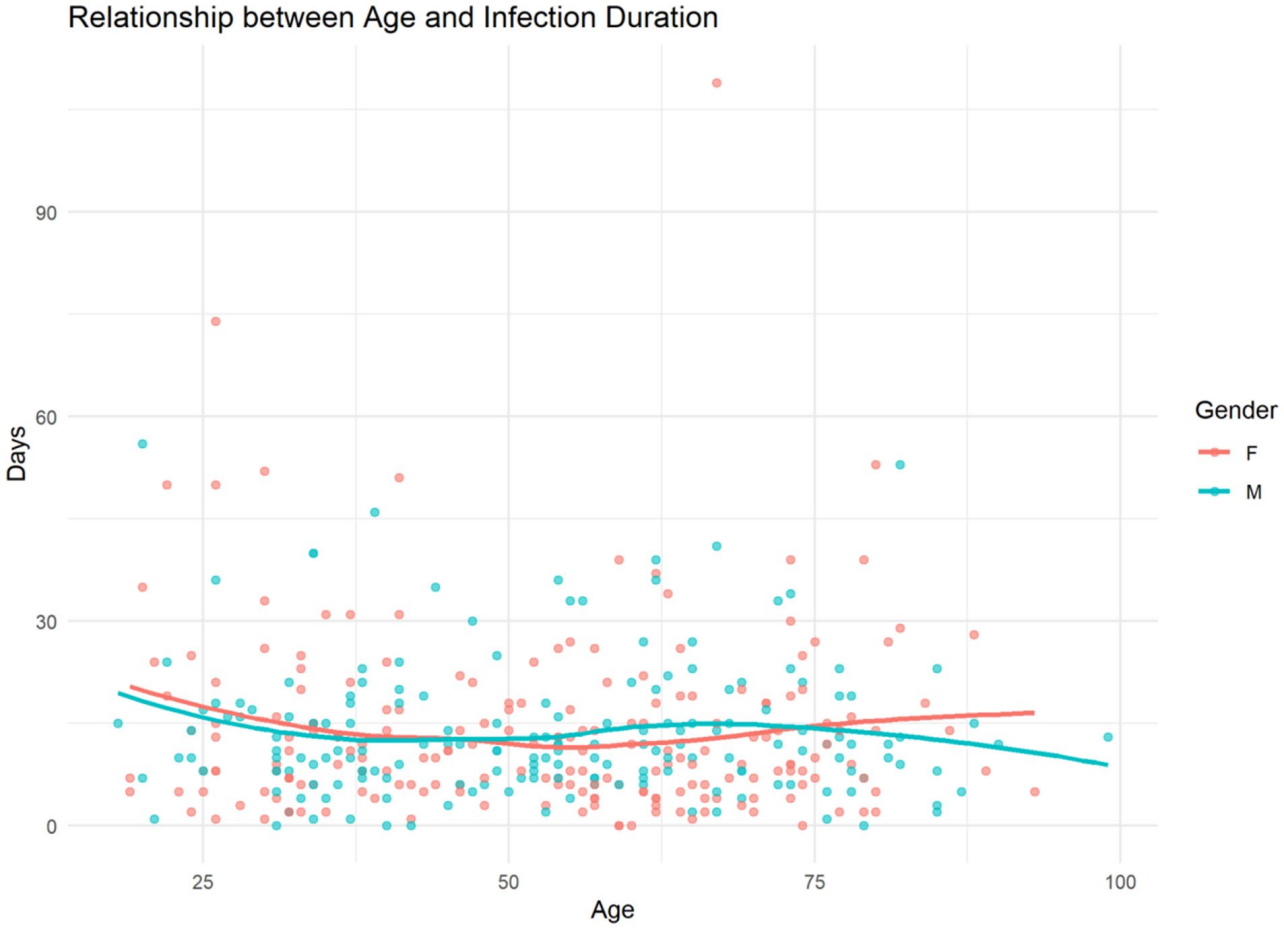
Relationship between age and infection duration. Scatter plot illustrates the relationship between patient age and the duration of healthcare-associated infection, measured in days. Each data point represents an individual case, color-coded by sex (red for female, blue for male). Trend lines for each sex suggest minimal variation in infection duration across age groups, with no marked difference observed between male and female patients.

When analyzing the duration of healthcare-associated infections in relation to patient age, no significant correlation was observed between the two factors. The trend lines for both sexes are practically parallel, suggesting that neither age nor sex had a relevant influence on the duration of the infection as shown in Figure 2.

### 3.3 Non-linear dynamics of monthly UTI incidence

**Figure 3:**
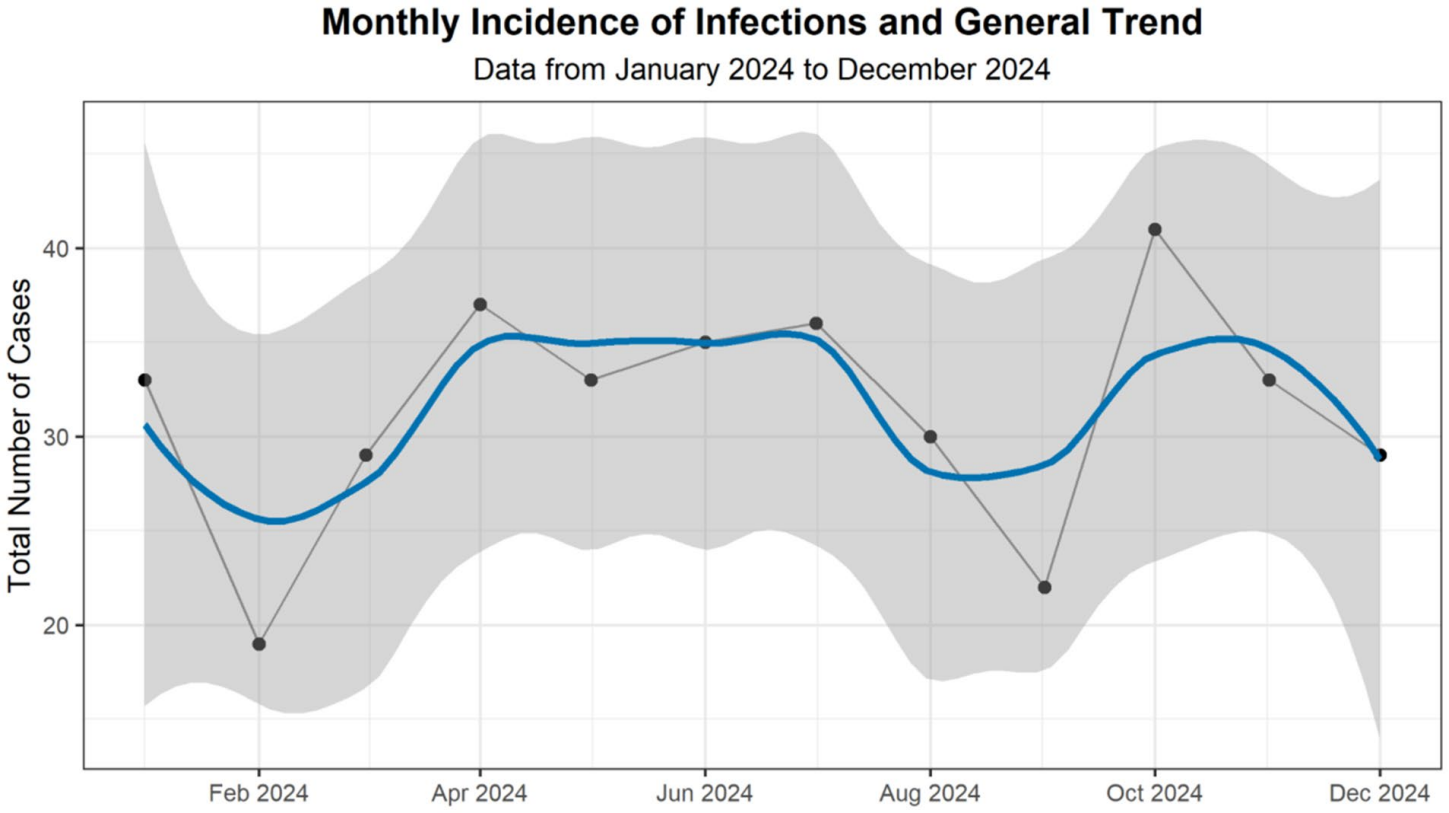
Monthly incidence of infections and general trend. Monthly incidence of urinary tract infections and temporal trend from January to December 2024. Black dots indicate the total number of cases recorded per month. The solid blue line represents a trend smoothed using LOESS (interval = 0.5), and the shaded gray area indicates the 95% confidence interval. The figure illustrates the descriptive temporal variation in case counts, without implying seasonality or causal associations.

Analysis of the monthly incidence of urinary tract infections (UTIs) from January to December 2024 showed temporal variability with non-uniform fluctuations throughout the study period (Figure 3). After a reduction in February, the number of cases increased during the following months, reaching a local peak in April, followed by a relatively stable phase until the beginning of summer. A subsequent decline was observed toward the end of summer, with a transient increase in October, which represented the highest monthly number recorded. The incidence then decreased toward the end of the year.

It is important to note that these observations reflect descriptive temporal variation rather than a defined seasonal pattern, and no causal or environmental factors were inferred from this analysis. The observed fluctuations primarily illustrate changes in the monthly case burden and support epidemiological surveillance and workload assessment, rather than etiological attribution.

### 3.4 Distribution of cases by treating medical service

**Figure 4:**
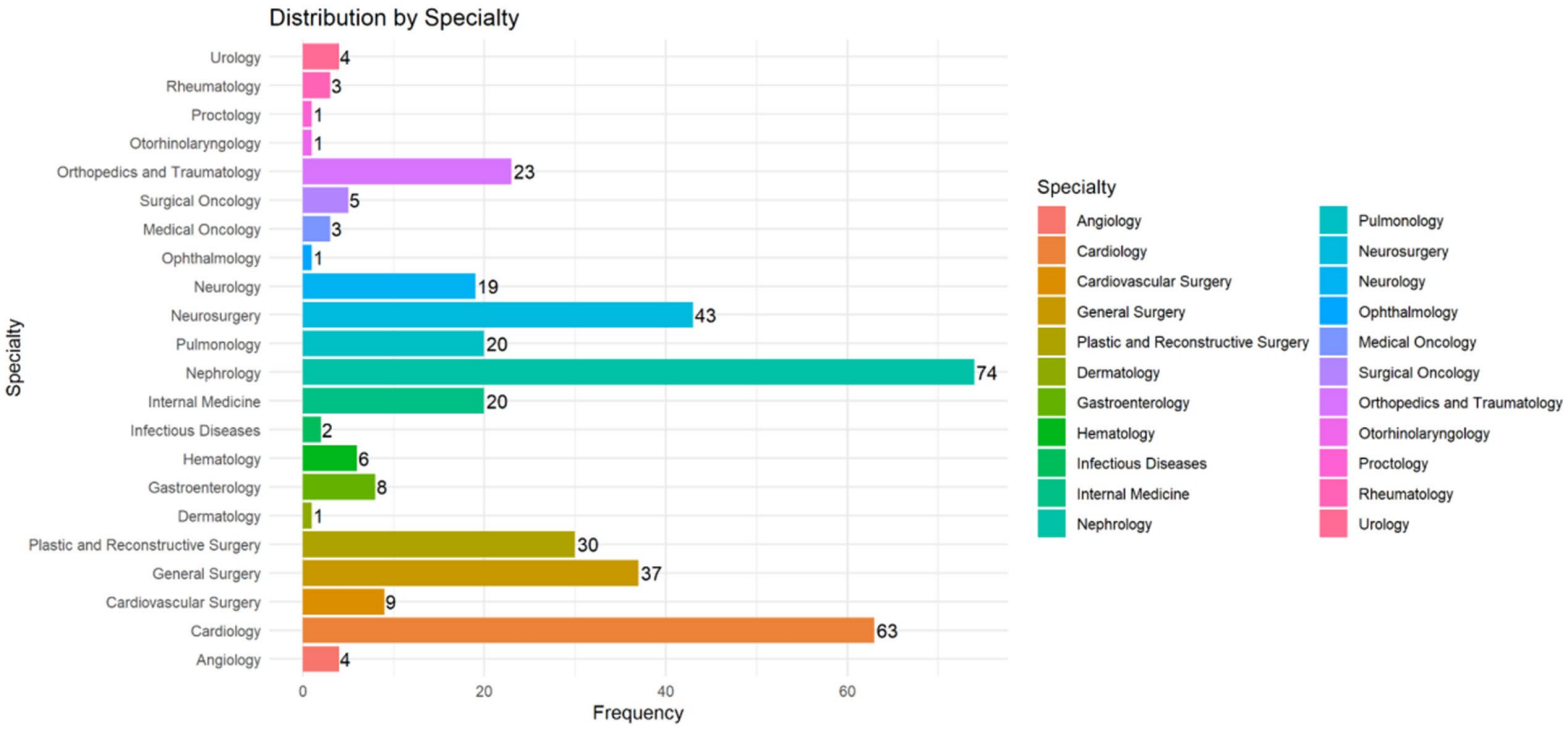
HAUTIs by medical service. Horizontal bar chart showing the distribution of patients by medical specialty. Each specialty is represented with a distinct color and corresponding frequency, with Nephrology (*n* = 74), Cardiology (*n* = 63), and Neurosurgery (*n* = 43) accounting for the highest number of cases.

The distribution of healthcare-associated infection cases by medical service is presented in Figure 4. The specialties with the highest number of patients were Nephrology (*n* = 74), Cardiology (*n* = 63), and Neurosurgery (*n* = 43), followed by General Surgery (*n* = 37) and Plastic and Reconstructive Surgery (*n* = 30). These five areas accounted for more than 60% of the recorded cases. Other specialties such as Dermatology, Proctology, and Ophthalmology reported considerably lower frequencies (*n* = 1 each), suggesting lower exposure or association with risk factors for this type of infection in those clinical contexts. Indeed, these results offer a relevant perspective on the hospital areas with the highest concentration of cases and could guide targeted prevention strategies.

### 3.5 Microbiological profile

Etiologic agents were identified in 120 of the 376 UTI cases included in the study (31.9%), corresponding to cases in which microbiological cultures were performed, and a documented isolate was obtained. The remaining cases were diagnosed based on clinical criteria and surveillance definitions, but no etiologic agent was recorded in the database, either due to negative cultures, lack of samples for culture, or prior antimicrobial exposure.

Among the 120 identified isolates, bacteria constituted the majority (*n* = 85, 70.8%). *Escherichia coli* was the most prevalent pathogen, representing 35.8% of all isolates (*n* = 43). Other frequently isolated Gram-negative bacteria were *Klebsiella pneumoniae* (*n* = 13, 10.8%) and *Pseudomonas aeruginosa* (*n* = 7, 5.8%). Fungal isolates accounted for 29.2% of cases (*n* = 35), with *Candida albicans* being the most common species (*n* = 19, 15.8%), followed by *Candida glabrata* (*n* = 8, 6.7%) and *Candida tropicalis* (*n* = 6, 5.0%). Detailed results are shown in Table 1.

**Table 1:** Etiologic agents.

Microorganism	*n* (%)
Isolated bacteria	85 (70.83)
*Acinetobacter baumannii*	1 (0.83)
*Aeromonas hydrophila*	1 (0.83)
*Citrobacter freundii*	1 (0.83)
*Coagulase-negative Staphylococcus (others)*	1 (0.83)
*Enterobacter cloacae complex*	1 (0.83)
*Enterococcus faecalis*	2 (1.67)
*Enterococcus faecium*	2 (1.67)
*Escherichia coli*	43 (35.83)
*Klebsiella aerogenes*	1 (0.83)
*Klebsiella oxytoca*	1 (0.83)
*Klebsiella pneumoniae*	13 (10.83)
*Klebsiella pneumoniae subsp. pneumoniae*	2 (1.67)
*Morganella morganii*	2 (1.67)
*Proteus mirabilis*	2 (1.67)
*Providencia rettgeri*	1 (0.83)
*Pseudomonas aeruginosa*	7 (5.83)
*Serratia marcescens*	1 (0.83)
*Staphylococcus aureus*	1 (0.83)
*Staphylococcus haemolyticus*	1 (0.83)
*Streptococcus agalactiae*	1 (0.83)
Isolated fungi	35 (29.17)
*Candida albicans*	19 (15.83)
*Candida glabrata*	8 (6.67)
*Candida tropicalis*	6 (5.00)
*Other Candida species*	1 (0.83)
*Trichosporon asahii*	1 (0.83)

Percentages are calculated based on the total number of isolates (120).

### 3.6 Antibiotic sensitivity and resistance

**Figure 5:**
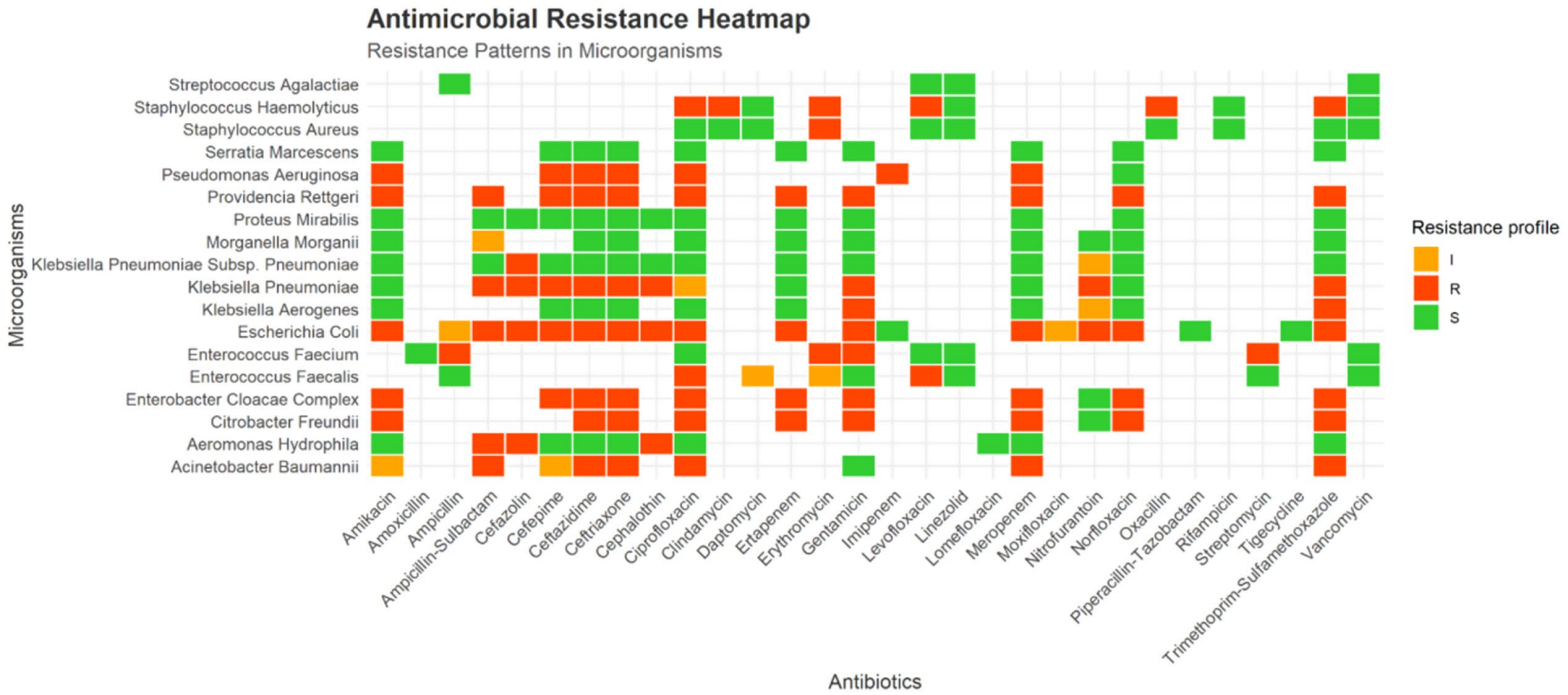
Antimicrobial resistance heatmap. Heatmap illustrating antimicrobial susceptibility profiles of clinical pathogens based on automated AST interpreted according to CLSI criteria. Susceptibility results are categorized as resistant (red), intermediate (orange), or susceptible (green). The figure provides a descriptive overview of resistance patterns across tested organisms and antimicrobial agents.

Antimicrobial susceptibility testing revealed heterogeneous resistance patterns across the identified pathogens (Figure 5). Several Gram-negative organisms exhibited high levels of non-susceptibility to multiple antimicrobial classes. Notably, *Providencia rettgeri* demonstrated resistance to all antibiotics included in the testing panel. High resistance rates were also observed among *Acinetobacter baumannii* isolates, including reduced susceptibility to carbapenems.

In contrast, *Klebsiella pneumoniae* and *Escherichia coli* isolates showed comparatively lower resistance to carbapenems, remaining largely susceptible according to CLSI interpretive criteria. Among Gram-positive organisms, vancomycin and linezolid, maintained activity against the isolates for which susceptibility testing was performed. Overall, these findings highlight substantial variability in antimicrobial susceptibility profiles among pathogens.

## 4 Discussion

This study provides an epidemiological and microbiological overview of UTIs in a tertiary-care hospital in Mexico. Most cases (80.6%) were healthcare-associated, primarily affecting middle-aged and elderly adults, consistent with earlier reports (Pasillas Fabian et al., 2021; Medina and Castillo-Pino, 2019). In this regard, in 2023 a study, reported that between 60 and 70% of Healthcare Associated Infections are associated with biofilms and 40% with catheter-associated urinary tract infections, which are the most common HAIs worldwide (Mengistu et al., 2023).

The bimodal incidence peaks in April and October indicate a complex, non-seasonal hospital dynamic, possibly reflecting variations in catheter use, patient turnover, or adherence to infection control. These non-linear patterns are rarely described in healthcare-associated infections, although a causal relationship has not been established. This finding could warrant further investigation, as each medical center has different characteristics, and the patient’s environment has been described as a risk factor (World Health Organization, 2022). Nephrology, Cardiology, and Neurosurgery emerged as the most affected services—consistent with high-risk patient populations exposed to invasive procedures and prolonged antibiotic therapy (Yang et al., 2022).

Microbiologically, *E. coli* remained dominant, aligning with global data, While the elevated fungal rate (29.2%) underscores the need to consider Candida-associated UTIs in catheterized or broad-spectrum antibiotic–treated patients (Sher et al., 2024; Reyna-Flores et al., 2013; Ballesteros-Monrreal et al., 2023). The antimicrobial resistance findings are alarming. The detection of pan-resistant *Providencia rettgeri* and multidrug-resistant *A. baumannii* underscores the urgency for local stewardship interventions (Iwata et al., 2022; Shin et al., 2018; Pal et al., 2024; Araya et al., 2023; Salmanov et al., 2019; Begum et al., 2013). Although carbapenems remain effective for most *E. coli* and *K. pneumoniae* isolates, vigilance is essential given rising resistance trends in Mexican tertiary hospitals (Wang et al., 2020; Song et al., 2024; Vachvanichsanong et al., 2020; Lee et al., 2017; Ernst et al., 2020; Bedenić et al., 2021).

In the global context, antibiotic resistance is one of the WHO’s major concerns, due to a notable increase in recent years (World Health Organization, 2025). One of the main causes of resistance is the discretionary use of drugs without adherence to local and regional empirical guidelines. The epidemiological surveillance of HAIs serves as a tool for measuring morbidity and mortality. Its usefulness is reflected in the successful implementation of infection prevention and control packages (World Health Organization, 2022).

Antibiotic resistance remains a threat to public health. In 2019 alone, CDC reported 5,000,000 AIAS worldwide and 1.27 million deaths associated with this type of infection. In the United States, approximately 2.8 million antimicrobial-resistant infections are reported each year, directly linked to 35,000 deaths in 2019 (World Health Organization, 2025; Centers for Disease Control and Prevention, 2025). The WHO, together with the governments of several countries associated with the United Nations (UN), has closed ranks with the aim of reducing and preventing antibiotic resistance. During the World Economic Forum (WEF 2019) in Davos, discussion was focused on acting effectively to counteract the rapid and massive spread of infectious diseases, including HAIs. This is due to 700,000 deaths per year worldwide from antibiotic-resistant bacteria. The authors estimate that by 2050, 10,000,000 deaths per year are expected (Huemer et al., 2020). The ability of bacteria to evolve and acquire antibiotic resistance has been a constant issue since antibiotics have been in use. However, in recent years, knowledge about the molecular mechanisms of resistance development has been enriched, promoting strategies for prevention, infection control, and rational use of antibiotics. These advances are derived from genomic technology, proteomics, structural biology, and have played a fundamental role in developing and updating new ways to detect resistance, how to manage complex cases, and the proper use of new antibiotics (Xia et al., 2016).

According to the World Health Organization, HAIs account for a substantial mortality burden worldwide. In the United States, approximately 100,000 deaths are attributed annually to HAIs, while European reports estimate close to 37,000 HAI-related deaths per year (Mazzeffi et al., 2021). Importantly, mortality associated with HAIs increases markedly in the presence of antimicrobial resistance, with evidence indicating that resistant infections can nearly double the risk of death (Mazzeffi et al., 2021). In terms of infection type, HAI-related mortality in the United States is distributed among ventilator-associated pneumonia (14.4%), central line–associated bloodstream infections (12%), surgical site infections (2.8%), CAUTIs (2.3%) (Mazzeffi et al., 2021). Beyond the clinical impact, HAIs impose a major economic burden; a 2013 analysis estimated that the annual cost of HAI management in the United States reaches approximately USD 10 billion (Katz, 2013).

Given the magnitude of the problem worldwide, urinary tract infections (UTIs) have been reviewed by various authors who focus their research on prevention strategies. One strategy that has proven successful in US hospitals is simple yet effective. The key to this strategy is its multidisciplinary approach, which includes infection prevention at all levels, proper Foley catheter insertion techniques, minimizing hospital stays, and even assessing the need for catheterization. A thorough analysis of these strategies reveals that they are replicable in our hospitals and that, more than funding, what is needed is raising awareness among medical staff. These strategies can be implemented beyond intensive care units (Van Decker et al., 2021; Trautner, 2010). The reality is simple: the presence of a catheter in the urethra has immediate effects, altering local immunity and predisposing the patient to infections. If inadequate aseptic and antiseptic techniques are added to this, the situation becomes unfavorable for the patient, especially if the catheter remains in place for a prolonged period. (Trautner, 2010) In the same context, the presence of a urethral catheter can cause symptoms simply due to its placement. Unfortunately, in many cases, urine cultures are taken unnecessarily, which can lead to misdiagnosis and inappropriate antibiotic use, generating resistance and affecting the quality of patient care (Chuang and Tambyah, 2021).

The focus on preventing and reducing urinary tract infections (UTIs) is not new. Programs addressing this problem have existed for more than 20 years. Sufficient evidence demonstrates that prevention programs remain largely the same (Lo et al., 2008; Yokoe et al., 2008) and include not only urinary tract infections but also other healthcare-associated infections. Two decades ago, researchers might have justified their inaction due to a lack of evidence (Trautner, 2010); however, there is now enough information to make informed decisions. It is clear that recommendations in the scientific literature do not always constitute clinical practice guidelines, as replicating a study can sometimes produce the opposite result (Westgeest et al., 2024). Determining whether patients require a specific antibiotic regimen cannot be considered a general rule, given the diversity of epidemiological scenarios. Resources can also vary depending on the geographical context, and even within the same country, significant epidemiological and budgetary differences may exist. However, the decision to choose the best antibiotic at the right time, the appropriate dose, and even whether or not to change a Foley catheter during hospitalization, is part of the reasoning process for healthcare professionals. As mentioned earlier, it is not necessary to resort to the most advanced technology or the most expensive antibiotics. What is needed is a focus on patients, their clinical course, and the critical and multidisciplinary thinking of healthcare professionals.

To prevent CAUTIs several changes should be applied to public health policy including continuing education for healthcare workers, improving good practices, and having better quality and availability in hand hygiene supplies. Public policy must guarantee a multidisciplinary approach to prevent infections and to act on time using appropriate antibiotic therapy to reduce resistance. It’s quite evident that all medical personnel should be aware of HAIs and understand the role of Hospital Epidemiological Surveillance Units in medical facilities (García-Quintero et al., 2023).

In the present report a similar trend was shown that has been reported in international literature. Resistance to antibiotics in HAUTIs has been under surveillance over the last years in our medical unit. Despite the efforts, increasing resistance patterns have been observed increasing morbidity, and economic health concerns.

Limitations include the single-center design, absence of clinical outcomes (e.g., mortality, length of stay), and lack of molecular typing for resistance mechanisms. Despite these constraints, the study offers robust surveillance data supporting improved infection-control, and antimicrobial strategies.

## 5 Conclusion

Healthcare-associated urinary tract infections are not minor problems, as the quality of patient care can be affected in various ways. One of the most worrying issues is the indiscriminate use of antibiotics and the increase in resistance. If we add to this the lack of reporting and inadequate management of cases, we could be facing a serious public health crisis. It is important that institutional programs promote initiatives that encourage the immediate reporting of healthcare-associated infections to epidemiological surveillance units, so that care for infected patients is multidisciplinary and leads to better outcomes.

## Data Availability

The raw data supporting the conclusions of this article will be made available by the authors, without undue reservation.
